# The Turkish validity and reliability of the Reward-Based Eating Drive (RED-13) Scale

**DOI:** 10.1371/journal.pone.0322097

**Published:** 2025-04-25

**Authors:** Ayten Yilmaz Yavuz, Canan Altinsoy, Merve Nur Toraman, Nazan Karabulut Musdal

**Affiliations:** 1 Department of Public Health Nursing, Faculty of Health Science, Recep Tayyip Erdogan University, Rize, Turkey; 2 Department of Nutrition and Dietetics, Faculty of Health Science, Recep Tayyip Erdoğan University, Rize, Turkey; 3 Training and Research Hospital, Recep Tayyip Erdogan University, Rize, Turkey; Ahvaz Jundishapur University: Ahvaz Jondishapour University of Medical Sciences, IRAN, ISLAMIC REPUBLIC OF

## Abstract

This paper established the Turkish validity and reliability of the Reward-based Eating Drive Scale (RED-13). This methodological study was conducted between January and June 2022. The study population consisted of all patients admitted to the dietary polyclinic of a hospital. The sample consisted of 500 participants. Data were collected using a personal information form and the Reward-Based Eating Drive Scale (RED-13). The data were analyzed using numbers, percentages, arithmetic means, standard deviations, correlation tests, t-tests, exploratory and confirmatory factor analyses, and Cronbach’s α reliability coefficients. The scale consists of 13 items and three subscales, explaining 66.1% of the total variance. The confirmatory factor analysis shows that x²/sd= 2.47, indicating an acceptable model. The goodness of fit indices are also acceptable (GFI = 0.99, AGFI = 0.99, CFI= 1.00, RMSEA = 0.08, and SRMR = 0.05). The total scale has a Cronbach’s α of 0.91. The first and second halves have Cronbach’s α values of 0.86 and 0.83, respectively. The scale has a Spearman-Brown coefficient of 0.85 and a Guttman Split-Half coefficient of 0.83. The scale has good internal consistency (reliability). The results indicate that the Reward-Based Eating Drive Scale (RED-13) is valid and reliable for the Turkish population.

**Trial registration:**

The study was registered on ClinicalTrials.gov (number: NCT05017506).

## Introduction

Obesity is characterised by an excessive accumulation or abnormal distribution of body fat (BF), which affects health and has become an escalating public health issue worldwide [1]. According to the WHO, one in eight people worldwide is living with obesity, and the prevalence of adult obesity has more than doubled since 1990. Similar to other countries around the world, the prevalence of obesity in Türkiye is increasing day by day. According to the latest data, the obesity rate (BMI ≥30 kg/m²) in adults and above is 31.5%, with a prevalence of 39.1% in women and 24.6% in men [2]. Estimates indicate that nearly 3.3 billion adults worldwide could have high BMI by 2035, up from 2.2 billion in 2020. This suggests an increase over 54% by 2035. The 2024 Global Burden of Disease study [3] reveals that over 56 million individuals, both adults and children, die annually due to diseases, injuries, or other health issues, resulting in a loss of 2.5 billion years of healthy life (measured as disability-adjusted life-years, or DALYs). Among these, approximately 42 million deaths and 1.6 billion DALYs are attributed to non-communicable diseases (NCDs). Notably, two-thirds of these NCD-related deaths and 40% of NCD DALYs arise from four major conditions: cancers (neoplasms), coronary heart disease, stroke, and diabetes. Each of these conditions is associated with, and accelerated by, overweight and obesity [4]. Eating patterns significantly contribute to these chronic diseases but are also a modifiable risk factor for prevention and management [5].Pleasure-based eating is becoming increasingly common with the spread of Western-style diets. In particular, easy access to palatable foods high in sugar, fat, and salt encourages people to adopt eating styles based on pleasure rather than energy needs [6]. Reward-based eating behaviors can enhance or suppress emotions, such as happiness, joy, stress, and anxiety [7]. If reward-based eating is used repeatedly in response to positive or negative emotions, it may lead to eating disorders. Therefore, researchers studying health behavior, nutrition, and metabolic health evaluate reward-based eating before, during, and after health behavior change interventions [8–10].

There are many scales in the literature assessing eating impulse and eating behavior, and most of them have been validated in the Turkish population. Eating Attitudes Test-26 (EAT-26), Three-Factor Eating Questionnaire (TFEQ) [11], Dutch Eating Behavior Questionnaire (DEBQ) [12], Yale Food Addiction Scale (YFAS) [13], Power of Food Scale (PFS) [14], Eating Disorder Examination Questionnaire (EDEQ-13) [15] are among these. When these measurement tools that have been studied in our country were examined, it was seen that either reward-based eating was not included at all or it was evaluated as a sub-dimension in some scales. There is no scale that assesses reward-based eating and the sub-dimensions of loss of control over eating, lack of satiety and preoccupation with food all at once.

Researchers use numerous scales to measure eating behaviors, such as reward-based eating, uncontrolled eating, food cravings, food addiction, restrictive eating, and binge eating. Each scale focuses on a different aspect of problematic eating behavior [16,17]. The Reward-based Eating Drive Scale (RED-13) (RED-9) was developed by Epel et al. (2014) to assess the full spectrum of reward-based eating. Although the RED-9 is short and easy to administer, it is unclear whether it assesses the full spectrum of reward-based eating [9,18]. Mason et al. (2014) expanded the 9-item “Reward Related Eating (RRE) Scale” to develop the RED-13. The RED-13 assesses three dimensions of reward-related eating, which are “loss of control over eating,” “lack of satiety,” and “preoccupation with food.” Research shows that a reduction in reward-based eating behavior may mediate the effect of obesity treatment on weight loss. The RED 13 has been shown as a valuable tool as a brief self-report measure that broadly captures the RRE spectrum [19].

This study aimed to establish the Turkish validity and reliability of the RED-13. This study will provide a database for interventions that address the effects of compulsive reward-based eating. RED-13 will help researchers and clinicians identify individuals who lack control over eating, do not feel full, and are constantly preoccupied with eating.

This study aims to test the validity and reliability of the Turkish version of the Reward-Based Eating Drive Scale (RED-13) in obese individuals. It is a finding supported by the literature that reward-based eating drives are more prominent in the obese population [20,21]; therefore, evaluating the psychometric properties of the scale in a population where reward-based eating is particularly relevant will enable the construct validity of the scale to be evaluated in a sample where it is most appropriate.

The Turkish adaptation of the RED-13 will provide clinicians and researchers with a valid tool to quantitatively assess pathological behaviours such as ‘loss of control over eating’, ‘lack of satiety’ and ‘preoccupation with food’. Furthermore, this study aims to provide a methodological basis for future intervention studies to understand the effect of reward mechanisms on eating behaviours in obese individuals.

## Materials and methods

### Research type

This study was a methodological study.

### Population and sample

This study was conducted in the Dietary Polyclinic of a Training and Research Hospital in the Eastern Black Sea Region of Türkiye between January and June 2022. Methodological studies use different approaches to determine sample size [20]. A common rule of thumb for scale development and adaptation studies is to have a sample size of <100 (very low), 101–199 (low), 200–299 (fair), 300–499 (good), 500–999 (very good), or >999 (excellent) [21]. The sample consisted of 500 adults who met the inclusion criteria and provided written and verbal consent to participate. The sample was divided into two groups for exploratory (n=250) and confirmatory (n=250) factor analyses. Reliability analyses were conducted on all participants [22]. The inclusion criteria were (1) being 18–65 years of age, (2) having a body mass index (BMI) of 30–39.9 kg/m^2^, (3) having at least a primary school degree, (4) being able to communicate verbally, and (5) volunteering. The exclusion criteria were (1) having a mental disorder, (2) not being able to communicate verbally, (3) not being able to read and understand Turkish, and (4) not being able to read and understand the data collection instruments.

The data were collected using a personal information form and the RED-13.

### Personal information form

The personal information form consisted of 11 items on sociodemographic and dietary characteristics.

### Reward-based Eating Drive (RED-13)

Ashley E. Mason and colleagues developed the nine-item Reward Related Eating (RRE) scale in 2014. They modified the scale and developed the 13-item Reward-based Eating Drive Scale (RED-13) in 2017. The items are rated on a five-point Likert-type scale (0=strongly disagree, 1=disagree, 2=neither agree nor disagree, 3=agree, 4=strongly agree). The total score ranges from 0 to 52, with higher scores indicating poor metabolic health [19]. The scale has three subscales: lack of satiety (LOS; items 7, 8, 9, and 11), (2) preoccupation with food (PO; items 5, 6, and 10), and (3) loss of control over eating (LOC; items 1, 2, 3, 4, 12, and 13) [19] The scale is short and reliable, capturing variability across reward-based feeding. Higher scores are associated with a greater appetite and craving for sweets. In the present study, the scale had a Cronbach’s α of 0.906.

### Research steps

Validity and reliability were examined in four steps.

### Language validity

Language validity was checked using back-translation, a standard method in scale adaptation research [23]. Three linguists translated the scale from English to Turkish. The researchers reviewed all three versions. Then, a Turkish linguist reviewed the scale. Two translators, who were native English speakers with experience in health terminology and linguistic and cultural aspects of the English language, translated the Turkish version back into English [24].

### Content validity

Ten experts (four public health nurses, one mental health and illness nurse, and five nutrition and dietetics specialists) with publications on eating disorders reviewed the scale for comprehensibility and relevance. Using a scale of 1–4, the experts employed the Davis technique to score the items’ relevance (content validity): 1 = not relevant, 2 = needs revision, 3 = relevant but needs minimal change, and 4 = very relevant. The Davis technique was used to determine the content validity ratio (CVR) [25].

### Pilot test

A pilot test was conducted with 30 adults who had similar characteristics to the main participants. The pilot test sample was not included in the main sample [23]. The pilot sample provided no negative feedback regarding readability, language, expression, clarity, and response time. The results showed that the scale items were understandable and relevant. In the pilot study, it was determined that the comprehensibility of the scale was sufficient and then it was applied to the whole sample.

### Data collection

Researchers briefed all adults on the research purpose and procedure. They obtained written informed consent from those who agreed to participate in the study. They then administered the scale to all participants. It took each participant 10–15 minutes to complete the scale. All participants found the scale items to be understandable and relevant. For test-retest reliability, a posttest was administered to 50 participants 20 minutes after the pretest [26]. The recruitment period spanned from February 1 to May 1, 2022.

### Data analysis

The researchers followed COSMIN (COnsensus-based Standards for the selection of health Measurement INstruments) [26]. The data were analyzed using the Statistical Package for Social Sciences (SPSS for Windows, version, 22) and LISREL 8.80 at a significance level of 0.05. Frequencies, percentages, and means were used for descriptive statistics. Content and construct validity were used to ensure the validity of the Turkish version of the scale. The Davis technique was used to determine the interrater agreement [27]. Exploratory factor analysis (EFA) and confirmatory factor analysis (CFA) were performed to determine the validity of the Turkish version of the scale. Exploratory factor analysis was used to determine the relationship between items and factors. The Kaiser-Meyer-Olkin (KMO) measure of sampling adequacy was employed to assess the suitability of the sample, whereas Bartlett’s test of sphericity was utilized to evaluate whether the correlations among the items were sufficient for factor analysis [28–30].

Confirmatory factor analysis was used to determine whether the items and subscales explain the original scale structure. The comparative fit index (CFI) for model validation was based on the chi-square test, degrees of freedom, root mean square error of approximation (RMSEA), goodness of fit index (GFI), and normed fit index (NFI) [20]. Item-total score analysis, Cronbach’s α coefficient, split-half analysis (Spearman-Brown and Guttman split-half values), and test-retest analysis were used to determine the internal consistency of the scale and its subscales (reliability). Pearson’s correlation coefficient was used for item-total score analysis.

### Ethical considerations

The researchers sent an email to Mason et al. (the developers of the scale) on September 13, 2021, requesting permission to adapt the scale to Turkish. The developers of the scale mistakenly gave permission for two adaptations [31]. However, they reviewed our back-translated version and permitted us to proceed to the following stages. An ethics committee then approved the study. Permission was obtained from the Institutional Review Board of the Recep Tayyip Erdogan University and the Provincial Directorate of Health (Date: 12/14/2021 & No: 2021/266). The researchers explained the purpose and procedure of the study to all adults. Informed consent was obtained from those who agreed to participate. The study was registered on ClinicalTrials.gov (number: NCT05017506).

## Results

The demographic characteristics of the participants are presented. More than half of the participants were women (69.2%) and married (77.2%). More than a quarter of the participants had a bachelor’s degree (29.2%). Less than half of the participants were housewives (35.8%). More than half of the participants had a chronic disease (56.0%). Half of the participants ate two meals a day (50.4%). Less than half of the participants never had snacks (44.8%). Most participants never ate at night (81.2%). Participants had a mean age of 42.19±12.17 years. They had a mean BMI of 34.34±2.92. They had a daily water intake of 1513.10±723.10 ml (Table 1).

**Table 1:** Sociodemographic characteristics of the participants.

		n (%)
**Gender**	Women	346 (69.2)
	Men	154 (30.8)
**Marital status**	Married	386 (77.2)
	Single	114 (22.8)
**Education (degree)**	Primary school	143 (28.6)
	Middle school	86 (17.2)
	High school	112 (22.49
	Bachelor’s	146 (29.2)
	Master’s	13 (2.6)
**Occupation**	Housewife	179 (35.8)
	Public official	83 (16.6)
	Worker	96 (19.2)
	Self-employed	93 (18.6)
	Retired	27 (5.4)
	Student	22 (4.4)
**Chronic disease**	Yes	280 (56.0)
	No	280 (44.0)
**Number of main meals**	1	9 (1.8)
	2	252 (50.4)
	3	239 (47.8)
**Snack habit**	Never	198 (39.6)
	1-3 times	284 (56.8)
	>3 times	18 (3.6)
**Eating at night**	Yes	40 (8.0)
	No	406 (81.2)
	Sometimes	54 (10.8)
**Continuous variables**		M±SD
Age		42.19±12.17
BMI		34.34±2.92
Daily water intake (ml)		1513.10±723.10

BMI, Body Mass Index; SD, Standart Deviation; M, Mean.

The items had CVR scores of 0.80 to 1.0. The total scale had a CVR of 0.98 and a KMO value of 0.879 (Bartlett *x*^2^=1678.491, p< 0.001).

**Fig 1:**
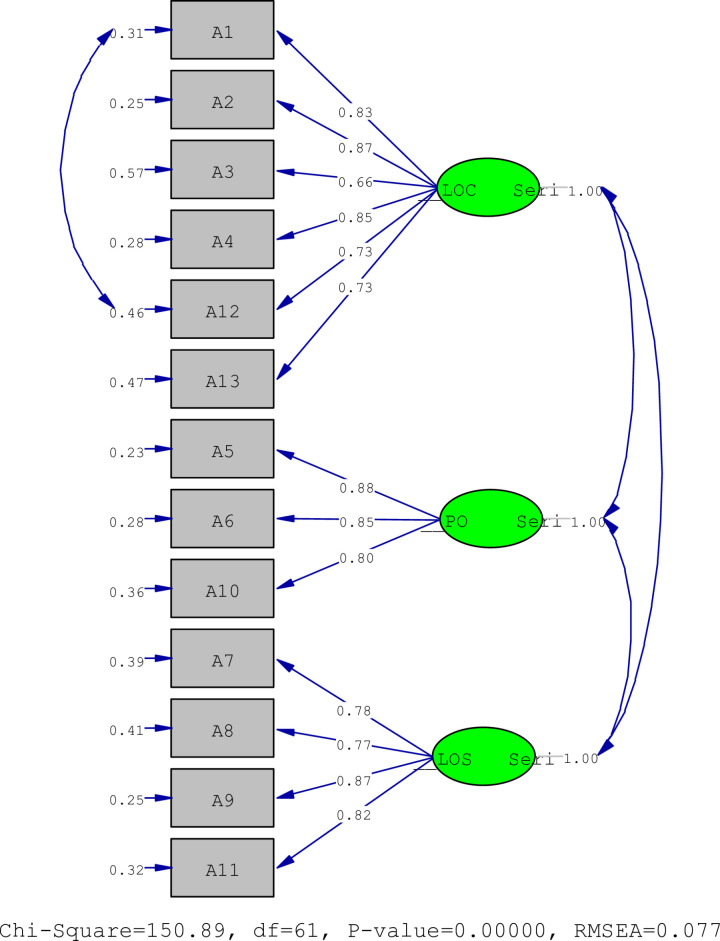
PATH diagram.

The results of the factor analysis are presented. The factor analysis results showed that the scale consisted of three subscales. All items had factor loadings of greater than 0.40. The subscales explained 66.062% of the total variance. The CFA results showed that the items had factor loadings of 0.56 to 0.87. The model was accepted as it was without any modification. The model had factor loadings of 0.66 to 0.88. All items had t values greater than 1.96 (12.09–20.40). The subscales “loss of control over eating,” “lack of satiety,” and “preoccupation with food” explained 28.007%, 16.477% and 21.578%, respectively (Table 2, Fig 1). Fig 1 presents the PATH diagram for the factor loadings of the subscales and items.

**Table 2:** Factor analysis results (three-factor original structure).

Item number	Items	Factor loading		
1	2	3
1.	I feel out of control in the presence of delicious food	.767	.088	.369
2.	When I start eating, I just can’t seems to stop	.770	.087	.353
3.	It is difficult for me to leave food on my plate	.560	.136	.109
4.	When it comes to foods I love, I have no willpower	.820	.084	.308
12.	Even though I am completely full, I feel myself continuing to consume certain foods even	.652	.421	.022
13.	If I like the food, I eat more than usual	.831	.183	.013
5.	I get so hungry that my stomach often looks like a bottomless pit	.144	.183	.867
6.	I don’t get full easily	.418	.184	.654
10.	I always feel hungry	.228	.469	.628
7.	It seems like most of my waking hours are preoccupied by thoughts about eating or not eating	.225	.555	.356
8.	There are days when I can’t think of anything else but food	.078	.806	.102
9.	I always have the thought of food/eating in my mind	.169	.829	.120
11.	I can’t get the idea of eating out of my mind, no matter how hard I try	.156	.789	.219
**Explained Variance (%)**		28.007	21.578	16.477
**Total Variance Explained (%)**		66.062		

It presents the scale’s values for *x*^2^/SD, GFI, AGFI, CFI, RMSEA, and SRMR values of 2.47, 0.99, 0.99, 1.00, 0.077, and 0.051, respectively (Table 3).

**Table 3:** Fit index values, normal and acceptable values.

Index	Normal value	Acceptable value	Result
*x*^2^/SD	<2	<5	2.47
GFI	>.95	>.90	.99
AGFI	>.95	>.90	.99
CFI	>.95	>.90	1.00
RMSEA	<.05	<.08	.077
SRMR	<.05	<.08	.051

*x*^2^, Chi-square; SD, Standart Deviation; GFI, Goodness of Fit Index; AGFI, Adjusted Goodness of Fit Index; CFI, Comparative Fit Index; RMSEA, Root Mean Square Error of Approximation; SRMR, Standardized Root Mean Square Residual.

It presents the Cronbach’s α values for the overall scale and its subscales. The scale had a Cronbach’s α of 0.906. The subscales “loss of control over eating”, “preoccupation with food” and “lack of satiety” had Cronbach’s α values of 0.879, 0.848, and 0.846, respectively. There was a positive correlation between pretest and posttest scores (r= 0.900, p<0. 001) (Table 4).

**Table 4:** Item-Total correlations and Cronbach’s α coefficients and split-half reliability values.

Items	n	Mean		SD.	Item-Total Correlations	Cronbach’s α (when item deleted)
1.	500	2.14		1.285	.668	.897
2.	500	1.89		1.225	.714	.895
3.	500	2.45		1.222	.498	.905
4.	500	2.28		1.247	.702	.896
5.	500	1.36		1.049	.621	.899
6.	500	1.61		1.092	.657	.898
7.	500	1.52		1.070	.597	.900
8.	500	1.14		.962	.545	.902
9.	500	1.25		.994	.624	.899
10.	500	1.43		1.060	.634	.899
11.	500	1.32		1.042	.617	.900
12.	500	2.23		1.175	.600	.900
13.	500	2.61		1.095	.603	.900
**Loss of control over eating Cronbach’s α**					.879	
**Preoccupation with food Cronbach’s α**					.848	
**Lack of satiety Cronbach’s α**					.846	
**Reward-based Eating Drive Scale Cronbach’s α**					.906	
					**Value**	**N of items**
**Cronbach’s α**			Half 1		.861	7^a^
			Half 2		.826	6^b^
			Total N of items		13	
**Correlation between forms**					.742	
**Spearman-Brown Coefficient**			Equal length		.852	
			Unequal length		.852	
**Guttman Split-Half Coefficient**					.834	

SD, Standard Deviation; a, The items are 1, 2, 3, 4, 5, and 6; b, The items are 7, 8, 9, 10, 11, 12, and 13.

It presents the split-half reliability values for the scale. The first half (seven items) had an internal consistency of 0.861, while the second half (six items) had an internal consistency of 0.826. The two halves had a correlation of 0.742, a Spearman-Brown coefficient of 0.852, and a Guttman Split-Half coefficient of 0.834 (Table 4).

## Discussion

In this study, we psychometrically evaluated a Turkish version of the reward-based eating drive (RED) questionnaire.

Content validity is a necessary step in scale validation studies. The total scale and each item should have an interrater agreement greater than 0.80 [32]. In the present study, the scale and each item had an interrater agreement greater than 0.80, indicating that there is a consensus among experts and that the scale measures the reward-based eating drive. In conclusion, the scale items can be used as they are. The Kaiser-Meyer Olkin (KMO) statistic for sampling adequacy was utilized to assess if the sample was sufficient, whereas Bartlett’s test of sphericity was applied to evaluate if the correlation among the items was suitable for factor analysis [33]. Bartlett’s test of sphericity (chi-square) should be <.05. The closer the KMO is to 1, the more suitable for factor analysis [21]. The KMO value obtained in this study showed that the sample size was adequate and also Bartlett’s test results show that the data are correlated with each other and suitable for factor analysis.

The explained variance ratio, an essential indicator of construct validity, should be above 40.0% in multidimensional scales. A higher ratio of variance explained results in stronger construct validity [33,34]. In the present study, the variance explained was greater than 50.0%, indicating strong construct validity. In addition, exploratory factor analysis is used to determine factor loadings. Each item should have a factor loading of greater than.50 [34,35]. The subscales of the original scale had factor loadings of 0.64 to 0.91 [19]. Our results pointed to similar factor loadings, indicating a strong factor structure. Researchers recommend that CFA follow EFA for validity and reliability [36,37]. An X^2^/df should be smaller than 5.0, while an RMSEA should be smaller than 0.08 [20,36,38]. Our results showed that the CFA values were consistent with the literature, indicating that the model was acceptable as it was. The X^2^/df value of this study is similar to the value of the original scale [19].

Cronbach’s α coefficient is used to assess whether the items are related to the subject matter to be measured. A Cronbach’s α coefficient should be between 0.60 and 1.00 [39]. Our results showed that the total scale and subscales had Cronbach’s α values greater than 0.70, indicating that the items are reliable enough to adequately measure reward-based eating drive in people with obesity. Split-half analysis is used to determine reliability. A split-half coefficient should be greater than 0.70 for reliability [39,40]. Our results showed that the split-half coefficients were greater than 0.70, indicating a strong correlation between the two halves. In other words, the results showed that the scale had good internal consistency. The findings suggest that the scale demonstrates strong reliability.

The Pearson product-moment correlation coefficient or test-retest reliability coefficient was used to examine the invariance of the scale after it was used twice at 20-minute intervals (pretest-posttest). Even if the test-retest correlation coefficient is satisfactory, it is recommended that the pretest and posttest scores be the same [41]. In this study, the test-retest reliability coefficients of the scale items were statistically significant. The fact that the items have the same pretest and posttest scores indicates they are clear and consistent.

Previous validation studies of the RED-13 scale—one in a general adult population and another in a student sample—have consistently confirmed its three-factor structure [42,43]. While these studies established the scale’s structural robustness across demographically distinct groups, our study extends this evidence by focusing specifically on an obese population, thereby addressing a critical gap in high metabolic-risk contexts. Similar to prior findings, we observed a strong positive correlation between higher RED-13 scores and elevated BMI, reinforcing the scale’s utility in linking reward-driven eating to obesity. Notably, the German study highlighted the need for clinical applications in conditions like type 2 diabetes and obesity [43]; our validation in an obese cohort directly responds to this call, demonstrating the scale’s relevance in populations where reward-based eating may exacerbate metabolic dysregulation.

Given these findings, high RED-13 scores should be interpreted not only as markers of reward-driven eating but also as clinically actionable indicators for obesity management. Elevated scores may reflect distinct neurobehavioral and psychological profiles that necessitate tailored interventions. High RED-13 scores indicate a greater tendency toward reward-based eating behaviors (e.g., emotional hunger, food cravings, and reward-seeking) [19]. Individuals with high scores may experience overactivation of brain reward circuits, difficulty in dietary adherence, or an increased risk of recurrent binge eating [44]. For example, individuals scoring high on the Loss of Control Over Eating subscale may struggle to regulate their eating behavior and may act in an “automatic pilot” mode while eating [45]. This may be associated with impulsivity, using food as a coping mechanism for stress, or excessive activation of neurobiological reward pathways. Cognitive behavioral therapy (CBT) and impulse control techniques can be beneficial in identifying and preventing triggers for binge episodes [46]. Individuals scoring high on the Lack of Satiety subscale may have difficulty perceiving physiological satiety signals or may continue eating for reward-seeking rather than actual fullness. In these cases, slow eating techniques and portion control training can be helpful. Additionally, adopting a fiber- and protein-rich diet may enhance satiety and regulate food intake [47]. Individuals with high scores on the Preoccupation with Food subscale may experience excessive cognitive engagement with food-related thoughts. For these individuals, mindfulness-based eating programs, activities that reduce cognitive preoccupation, and non-restrictive dietary approaches (e.g., Intuitive Eating) may be effective. These strategies can help diminish the perception of forbidden foods and promote a more balanced approach to eating [48].

Although the factor structure of the Turkish RED-13 scale is valid, it should not be ignored that cultural factors may profoundly affect the clinical and behavioral interpretation of the scale results. Cultural factors not only influence eating habits but also modulate how individuals perceive, report, and internalize reward-based eating tendencies [49]. In the Turkish context, traditional dietary practices, food-related social norms, the importance of communal eating, family dynamics, religious practices (e.g., mass iftar rituals during Ramadan), and gender roles (where women generally assume greater culinary responsibilities and are more engaged with food than men) may influence how individuals perceive and report reward-driven eating tendencies. For instance, the cultural emphasis on shared meals and hospitality might affect self-regulation in food consumption. Furthermore, the availability and preference for certain high-reward foods (e.g., sweets, pastries) in Turkish cuisine, along with the growing variety and consumption of processed foods [50], and the rapid access to particularly rewarding foods through the increasing use of online ordering applications, which surged during the pandemic [51], may contribute to reward-based eating behaviours. These cultural elements should be considered when interpreting the results, and future studies should further examine their impact through cross-cultural comparisons or qualitative assessments.

### Limitations and strengths

This study contributes to the growing field of personalized nutrition and behavioral therapy in weight management by validating the RED scale in a specific clinical population. The ability to rapidly and accurately assess reward-based eating in real-world settings enhances the practicality of dietary and behavioral interventions. By identifying individuals who may benefit from targeted approaches, this validation study strengthens the clinical utility of the RED scale, supporting timely identification and intervention strategies to optimize patient outcomes. To ensure rigor, all anthropometric measurements were conducted by trained dietitians. This eliminated the risk of self-reported bias in body weight and height. Participants were required to have at least a primary school education to ensure accurate comprehension of the survey items and reliable data collection. Additionally, individuals with diagnosed mental disorders were excluded to minimize potential confounding effects on reward-based eating behaviors.

However, this study has certain limitations that should be acknowledged. Additionally, the study focused on individuals with a BMI of 30–39.9 kg/m², meaning that the validity of the RED scale in individuals with lower or higher BMI remains to be explored. While this BMI range was selected due to its clinical relevance and the established relationship between reward-based eating and obesity, future studies should aim to validate the scale across a wider spectrum of BMI categories and non-clinical populations.

## Conclusions

In this study, a new measurement tool was introduced to our country by conducting the Turkish validity and reliability study of the “The Reward-based Eating Drive Scale (RED-13) developed by Ashley E. Mason. The RED-13 can be used to assess reward-based eating drive in people with obesity. Healthcare professionals and researchers use the RED-13 to identify eating drives in the Turkish population.

## References

[1] Mayoral LP-C, Andrade GM, Mayoral EP-C, Huerta TH, Canseco SP, Rodal Canales FJ, et al. Obesity subtypes, related biomarkers & heterogeneity. Indian J Med Res. 2020;151(1):11–21. doi: 10.4103/ijmr.IJMR_1768_17 32134010 PMC7055173

[2] Obezite. [cited 8 Mar 2025]. Available from: https://hsgm.saglik.gov.tr/tr/obezite.

[3] Global Burden of Disease 2021: Findings from the GBD 2021 Study | Institute for Health Metrics and Evaluation. [cited 9 Mar 2025]. Available from: https://www.healthdata.org/research-analysis/library/global-burden-disease-2021-findings-gbd-2021-study

[4] Obesity Atlas 2024 | World Obesity Federation Global Obesity Observatory. [cited 8 Mar 2025]. Available from: https://data.worldobesity.org/publications/?cat=22

[5] Shan Z, Wang F, Li Y, Baden MY, Bhupathiraju SN, Wang DD, et al. Healthy eating patterns and risk of total and cause-specific mortality. JAMA Intern Med. 2023;183(2):142–53. doi: 10.1001/jamainternmed.2022.6117 36622660 PMC9857813

[6] Lowe MR. Self-regulation of energy intake in the prevention and treatment of obesity: is it feasible? Obes Res. 2003;11 Suppl:44S–59S. doi: 10.1038/oby.2003.223 14569037

[7] Evers C, Adriaanse M, de Ridder DTD, de Witt Huberts JC. Good mood food. Positive emotion as a neglected trigger for food intake. Appetite. 2013;68:1–7. doi: 10.1016/j.appet.2013.04.007 23602962

[8] Forman EM, Butryn ML, Juarascio AS, Bradley LE, Lowe MR, Herbert JD, et al. The mind your health project: a randomized controlled trial of an innovative behavioral treatment for obesity. Obesity (Silver Spring). 2013;21(6):1119–26. doi: 10.1002/oby.20169 23666772 PMC3735809

[9] Mason AE, Epel ES, Aschbacher K, Lustig RH, Acree M, Kristeller J, et al. Reduced reward-driven eating accounts for the impact of a mindfulness-based diet and exercise intervention on weight loss: Data from the SHINE randomized controlled trial. Appetite. 2016;100:86–93. doi: 10.1016/j.appet.2016.02.009 26867697 PMC4799744

[10] Stevenson BL, Dvorak RD, Wonderlich SA, Crosby RD, Gordon KH. Emotions before and after loss of control eating. Eat Disord. 2018;26(6):505–22. doi: 10.1080/10640266.2018.1453634 29565734

[11] Küçükerdönmez Ö, Akder RN, Seçkiner S, Oksel E, Akpınar Ş, Köksal E. Turkish version of the “Three-Factor Eating Questionnaire-51” for obese individuals: a validity and reliability study. Public Health Nutr. 2021;24(11):3269–75. doi: 10.1017/S1368980021000574 33568253 PMC8314920

[12] Bozan N, Bas M, Asci FH. Psychometric properties of Turkish version of Dutch Eating Behaviour Questionnaire (DEBQ). A preliminary results. Appetite. 2011;56(3):564–6. doi: 10.1016/j.appet.2011.01.025 21277923

[13] Buyuktuncer Z, Akyol A, Ayaz A, Nergiz-Unal R, Aksoy B, Cosgun E, et al. Turkish version of the Yale Food Addiction Scale: preliminary results of factorial structure, reliability, and construct validity. J Health Popul Nutr. 2019;38(1):42. doi: 10.1186/s41043-019-0202-4 31822299 PMC6905049

[14] Ulker I, Ayyildiz F, Yildiran H. Validation of the Turkish version of the power of food scale in adult population. Eat Weight Disord. 2021;26(4):1179–86. doi: 10.1007/s40519-020-01019-x 33006077

[15] Esin K, Ayyıldız F. Validity and reliability of the Turkish version of the Eating Disorder Examination Questionnaire (EDE-Q-13): short-form of EDE-Q. J Eating Disord. 2022;10(1):1–9. doi: 10.1186/S40337-022-00628-4 PMC9281018 35836297

[16] Price M, Higgs S, Lee M. Self-reported eating traits: underlying components of food responsivity and dietary restriction are positively related to BMI. Appetite. 2015;95:203–10. doi: 10.1016/j.appet.2015.07.006 26162952

[17] Vainik U, Neseliler S, Konstabel K, Fellows LK, Dagher A. Eating traits questionnaires as a continuum of a single concept. Uncontrolled eating. Appetite. 2015;90:229–39. doi: 10.1016/j.appet.2015.03.004 25769975

[18] Mason AE, Laraia B, Daubenmier J, Hecht FM, Lustig RH, Puterman E, et al. Putting the brakes on the “drive to eat”: pilot effects of naltrexone and reward-based eating on food cravings among obese women. Eat Behav. 2015;19:53–6. doi: 10.1016/j.eatbeh.2015.06.008 26164674 PMC4644449

[19] Mason AE, Vainik U, Acree M, Tomiyama AJ, Dagher A, Epel ES, et al. Improving assessment of the spectrum of reward-related eating: The RED-13. Front Psychol. 2017;8:795. doi: 10.3389/fpsyg.2017.00795 28611698 PMC5447741

[20] Sencan H. Reliability and Validity in Social and Behavioral Measures. Ankara: Seckin Publishing; 2005.

[21] Karagoz Y. SPSS and AMOS 23 Applied Statistical Analysis. First. Nobel Publication; 2018.

[22] DeVellis R, Thorpe C. Scale Development: Theory and Applications. Los Angeles: Sage Publications; 2017.

[23] Çapık C, Gözüm S, Aksayan S. Intercultural scale adaptation stages, language and culture adaptation: updated guideline. Florence Nightingale Hemşirelik Dergisi. 2018:199–210. doi: 10.26650/fnjn397481

[24] Sousa VD, Rojjanasrirat W. Translation, adaptation and validation of instruments or scales for use in cross-cultural health care research: a clear and user-friendly guideline. J Eval Clin Pract. 2011;17(2):268–74. doi: 10.1111/j.1365-2753.2010.01434.x 20874835

[25] Davis LL. Instrument review: getting the most from a panel of experts. Appl Nurs Res. 1992;5(4):194–7. doi: 10.1016/s0897-1897(05)80008-4

[26] Mokkink LB, Terwee CB, Patrick DL, Alonso J, Stratford PW, Knol DL, et al. The COSMIN checklist for assessing the methodological quality of studies on measurement properties of health status measurement instruments: an international Delphi study. Qual Life Res. 2010;19(4):539–49. doi: 10.1007/s11136-010-9606-8 20169472 PMC2852520

[27] Ercan I, Kan I. Reliability and validity in the scales. J Uludag Univ Faculty Med. 2004;30(1):211–6.

[28] Kaiser HF. A second generation little jiffy. Psychometrika. 1970;35(4):401–15. doi: 10.1007/bf02291817

[29] Simsek O. Introduction to structural equation modeling, basic principles and LISREL applications. İstanbul: Ekinoks Education Consultancy Service; 2010.

[30] Zamanzadeh V, Ghahramanian A, Rassouli M, Abbaszadeh A, Alavi-Majd H, Nikanfar A-R. Design and implementation content validity study: development of an instrument for measuring patient-centered communication. J Caring Sci. 2015;4(2):165–78. doi: 10.15171/jcs.2015.017 26161370 PMC4484991

[31] Saruhan N. Validity and reliability study of reward-related eating scale into Turkish. Master Thesis, Istanbul Aydin University Institute of Graduate Studies. 2021.

[32] Polit DF, Beck CT, Owen SV. Is the CVI an acceptable indicator of content validity? Appraisal and recommendations. Res Nurs Health. 2007;30(4):459–67. doi: 10.1002/nur.20199 17654487

[33] Boateng GO, Neilands TB, Frongillo EA, Melgar-Quiñonez HR, Young SL. Best practices for developing and validating scales for health, social, and behavioral research: a primer. Front Public Health. 2018;6:149. doi: 10.3389/fpubh.2018.00149 29942800 PMC6004510

[34] Finch H. Exploratory Factor Analysis. SAGE Publications; 2019.8

[35] Hair JF, Black WC, Babin BJ, Anderson RE. Multivariate data analysis. 8th ed. 2019 [cited 9 Mar 2025]. Available from: www.cengage.com/highered

[36] Brown T. Confirmatory factor analysis for applied research. 2nd ed. The Guilford Press; 2015.

[37] Xia Y, Yang Y. RMSEA, CFI, and TLI in structural equation modeling with ordered categorical data: the story they tell depends on the estimation methods. Behav Res Methods. 2019;51(1):409–28. doi: 10.3758/s13428-018-1055-2 29869222

[38] Marsh HW, Guo J, Dicke T, Parker PD, Craven RG. Confirmatory Factor Analysis (CFA), Exploratory Structural Equation Modeling (ESEM), and Set-ESEM: Optimal Balance Between Goodness of Fit and Parsimony. Multivariate Behav Res. 2020;55(1):102–19. doi: 10.1080/00273171.2019.1602503 31204844

[39] Nunnally J. Psychometric Theory. New York: McGraw-Hill Publisher; 2010.

[40] Chakrabartty S. Best Split-Half and Maximum Reliability. 2013.

[41] Noble S, Scheinost D, Constable RT. A decade of test-retest reliability of functional connectivity: a systematic review and meta-analysis. Neuroimage. 2019;203:116157. doi: 10.1016/j.neuroimage.2019.116157 31494250 PMC6907736

[42] Sutton CA, L’Insalata AM, Fazzino TL. Reward sensitivity, eating behavior, and obesity-related outcomes: a systematic review. Physiol Behav. 2022;252:113843. doi: 10.1016/j.physbeh.2022.113843 35577107

[43] Herhaus B, Barlang M, Berth H, Vainik U, Epel ES, Tomiyama AJ, et al. Factor structure and psychometric properties of the German version of the Reward-based Eating Drive scale. 2024. [cited 8 Mar 2025]. doi: 10.21203/rs.3.rs-5268393/v1

[44] Frank GKW. Altered brain reward circuits in eating disorders: chicken or egg?. Curr Psychiatry Rep. 2013;15(10):396. doi: 10.1007/s11920-013-0396-x 23963630 PMC3888645

[45] Conceição EM, Moreira CS, de Lourdes M, Ramalho S, Vaz AR. Exploring correlates of loss of control eating in a nonclinical sample. Front Psychol. 2022;12:787558. doi: 10.3389/fpsyg.2021.787558 35222152 PMC8874330

[46] Kessler RM, Hutson PH, Herman BK, Potenza MN. The neurobiological basis of binge-eating disorder. Neurosci Biobehav Rev. 2016;63:223–38. doi: 10.1016/j.neubiorev.2016.01.013 26850211

[47] Blundell J, de Graaf C, Hulshof T, Jebb S, Livingstone B, Lluch A, et al. Appetite control: methodological aspects of the evaluation of foods. Obes Rev. 2010;11(3):251–70. doi: 10.1111/j.1467-789X.2010.00714.x 20122136 PMC3609405

[48] Lydecker JA, Simpson L, Smith SR, White MA, Grilo CM. Preoccupation in bulimia nervosa, binge-eating disorder, anorexia nervosa, and higher weight. Int J Eat Disord. 2022;55(1):76–84. doi: 10.1002/eat.23630 34713460 PMC8963447

[49] Enriquez JP, Archila-Godinez JC. Social and cultural influences on food choices: a review. Crit Rev Food Sci Nutr. 2022;62(13):3698–704. doi: 10.1080/10408398.2020.1870434 33427479

[50] Ülker İ, Çamli A. Ultra İşlenmiş Besin Tüketimi Hedonik Açlığı Etkiler Mi?. J Nutr Diet. 2024;52(1):68–76. doi: 10.33076/2024.bdd.1839

[51] Özbek A, Akyüz Kantarci Ö. Evaluation of online food ordering service platforms using MCDM methods. Int Sci Voc Stud J. 2024;8(2):178–91. doi: 10.47897/bilmes.1578941

