# Correction: The Turkish validity and reliability of the Reward-Based Eating Drive (RED-13) Scale

**DOI:** 10.1371/journal.pone.0333804

**Published:** 2025-10-06

**Authors:** Ayten Yilmaz Yavuz, Canan Altinsoy, Merve Nur Toraman, Nazan Karabulut Musdal

In the Trial registration subsection of the Abstract and Ethical considerations subsection of the Materials and methods, the clinical trial link and registration number are listed incorrectly. The correct clinical trial link is: https://clinicaltrials.gov/study/NCT05554250. The correct clinical trial registration number is: NCT05554250.
